# Quantitative Analysis of l-Arginine, Dimethylated Arginine Derivatives, l-Citrulline, and Dimethylamine in Human Serum Using Liquid Chromatography–Mass Spectrometric Method

**DOI:** 10.1007/s10337-018-3520-6

**Published:** 2018-04-21

**Authors:** Mariusz G. Fleszar, Jerzy Wiśniewski, Małgorzata Krzystek-Korpacka, Błażej Misiak, Dorota Frydecka, Joanna Piechowicz, Katarzyna Lorenc-Kukuła, Andrzej Gamian

**Affiliations:** 1 Department of Medical Biochemistry, Wroclaw Medical University, ul. Chalubinskiego 10, 50-368 Wroclaw, Poland; 2 Shimadzu Center for Advanced Analytical Chemistry, University of Texas at Arlington, Arlington, TX USA; 3 Department of Psychiatry, Wroclaw Medical University, 10 Pasteur Street, 50-367 Wroclaw, Poland; 4 Department of Genetics, Wroclaw Medical University, 1 Marcinkowski Street, 50-368 Wroclaw, Poland; 5 Wroclaw Research Center EIT+, Wroclaw, Poland

**Keywords:** Liquid chromatography–mass spectrometry, Amino acid derivatization, Nitric oxide, Asymmetric dimethylarginine, Citrulline, Dimethylamine

## Abstract

**Abstract:**

Nitric oxide (NO) is a small molecule involved in the regulation of many physiological processes. It plays a crucial role in the regulation of nervous system, immune and inflammatory responses, and blood flow. NO is synthesized by nitric oxide synthase (NOS) during two-step oxidation of l-arginine to l-citrulline. Intermediates and derivatives of NO metabolism, such as l-arginine, l-citrulline, asymmetrical dimethylarginine (ADMA), symmetrical dimethylarginine (SDMA), and dimethylamine (DMA), are investigated as potential biomarkers. In this article, we present a novel analytical method that allowed for simultaneous analysis of l-arginine, ADMA, SDMA, l-citrulline, and DMA, in a single-step extraction and derivatization using benzoyl chloride. In brief, aliquots of serum were mixed with internal standard solution mixture (50 µM D6-DMA, 20 µM D7-ADMA, and 100 µM D7-arginine) and 0.025 M borate buffer, pH 9.2 (10:1:5). The derivatization process was performed at 25 °C for 5 min using 10% benzoyl chloride. A reverse phase column was used for chromatographic separation. Quantitation was performed using following ions (*m/z*): 279.1457, 286.1749, 307.1717, 314.2076, 280.1297, 150.0919, and 156.1113 for l-arginine, D7-arginine, ADMA, SDMA, D7-ADMA, l-citrulline, DMA, and D6-DMA, respectively. The method was validated, and its assay linearity, accuracy and precision, recovery, and limits of detection (1.7 µM l-arginine, 0.03 µM ADMA, 0.02 µM SDMA, 0.36 µM l-citrulline, 0.06 µM DMA) and quantification (3.2 µM l-arginine, 0.08 µM ADMA, 0.05 µM SDMA, 1.08 µM l-citrulline, 0.19 µM DMA) were determined. The method is sensitive, reliable, repeatable, and reproducible. It can be applied in the routine clinical/diagnostic laboratory.

**Graphical abstract:**

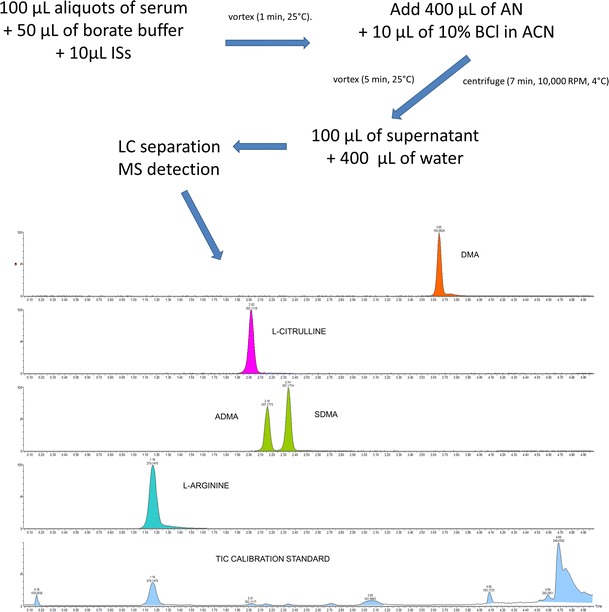

## Introduction

Nitric oxide (NO) is a signaling molecule involved in the regulation of many biological processes, such as vascular and airway tone regulation, angiogenesis, inflammatory response and immunity, nerve signal transmission, and insulin synthesis [1]. Nitric oxide is produced during the two-step oxidation of l-arginine to l-citrulline, catalyzed by nitric oxide synthase (NOS). There are three isoforms of the enzyme, two of which are calcium dependent and expressed constitutively—neuronal and endothelial NOS (nNOS/NOS I and eNOS/NOS III, respectively)—and one inducible and calcium independent (iNOS/NOS II). Nitric oxide synthase activity is regulated by both negative feedback [2, 3] and availability of l-arginine [4]. l-Arginine is a semi-essential amino acid obtained either from the diet or synthesized endogenously from L-citrulline. l-Citrulline, in turn, can be derived from ornithine in the catabolism of proline or glutamine and glutamate, or from l-arginine via arginine–citrulline pathway. l-Citrulline can also be obtained during the degradation of asymmetric dimethylarginine (ADMA), the process catalyzed by dimethylarginine dimethylaminohydrolase (DDAH), yielding dimethylamine (DMA) as a coproduct [5]. Asymmetric dimethylarginine is constitutively synthesized during the turnover of methylated proteins. In addition to being a potential source of l-citrulline, ADMA acts as endogenous inhibitor of several NOS isoforms, including nNOS, and regulates l-arginine availability by controlling the transport of amino acid across the cell membranes [6–8]. Symmetric dimethylarginine (SDMA)—ADMA regioisomer—is considered only as a weak intermediate NOS inhibitor and is competing with l-arginine for transport across cell membranes [9]. SDMA has been recognized as an effective marker of renal function [10]. There are also growing number evidences supporting participation of SDMA in the development of inflammation and atherosclerosis.

Disturbances in NO metabolism accompanied by abnormal concentrations of its intermediates are implicated in the pathogenesis of hypertension, hypercholesterolemia, diabetes, cardiovascular and kidney diseases, schizophrenia, Alzheimer’s disease, intestinal failure, and cancer [1]. Therefore, l-arginine, l-citrulline, ADMA, SDMA, and DMA have gained attention as potential disease markers and/or goals of newly designed therapeutic strategies. Therefore, the concomitant determination of their exact concentration that enables the assessment of their mutual correlations is desired. Moreover, simultaneous assessment of a panel of intermediates and derivatives of NO metabolism is a time- and cost-saving approach, in which the amount of biological material, like human blood, is substantially reduced rendering the method more easily acceptable by patients. However, to the best of our knowledge, there are no methods have been reported for simultaneous measurement of l-arginine, l-citrulline, ADMA, SDMA, and DMA via liquid chromatography–mass spectrometry (LC–MS). There are only a few reported cases of DMA analysis in human biological material, including inter alia high-performance liquid chromatography (HPLC) coupled with fluorescence detection [11] and gas–liquid chromatography–mass spectrometry (GLC–MS) [12]. In turn, already published methods for separate quantitative analyses of ADMA and SDMA might not be easily accessible [13–19] as they require triple quadrupole mass spectrometers (operating in multiple reaction monitoring—MRM—mode). Other reported methods of ADMA and SDMA analysis without derivatization utilized hydrophilic liquid interaction chromatography (HILIC) which is suitable for small polar molecules. Some of those methods are suboptimal because they utilize trifluoroacetic acid (TFA). TFA is a known signal suppressor in electrospray ionization source (ESI), thereby decreasing method’s sensitivity [17]. Also, the use of HILIC methods usually involves longer column equilibration times, and HILIC column tends to show less reproducibility or durability compared with standard reversed-phase columns. In addition, the derivatization enables the analysis of molecules with *m/z* below 100, the determination of which on the QTOF mass spectrometers is problematic. Here, we present an efficient and sensitive method for the simultaneous analysis of l-arginine, l-citrulline, ADMA, SDMA, and DMA in human serum using benzoyl chloride as a derivatization reagent, analyzed by reversed-phase chromatography and by means of Xevo G2 XS Quadrupole TOF MS (Waters) with ESI ion source.

## Materials and Methods

### Chemicals

Benzoyl chloride (BCl), hydrochloride salts of unlabeled dimethylamine (D0-DMA), hexadeutero-dimethylamine (D6-DMA, declared as 99 at.% 2H), l-arginine, SDMA, ADMA, l-citrulline, and sodium tetraborate were procured from Sigma-Aldrich (Poznan, Poland). Isotope-labeled l-arginine:HCl (D7-arginine, 98%) and asymmetric dimethylarginine (2,3,3,4,4,5,5-D7-ADMA, 98%) were obtained from Cambridge Isotope Laboratories (Tewksbury, MA, USA). Methanol, acetonitrile, water, and formic acid were acquired from Merck Millipore (Warsaw, Poland), and leucine–enkephalin was obtained from Waters (Milford, MA, USA).

### Preparation of Standard Calibration Curves and Human Serum Samples

Calibration standards and serum samples were prepared in the same manner. The following concentrations of calibrators, in water, were used: 5, 12.5, 25, 50, 100, 150, 200, 250 µM for l-arginine, 0.05, 0.13, 0.25, 0.5, 1.0, 1.5, 2, and 2.5 µM for ADMA and SDMA, and 1, 2.5, 5, 10, 20, 30, 40, and 50 µM for l-citrulline, and 0.14, 0.35, 0.7, 1.4, 2.8, 4.2, 5.6, 7.0 µM for DMA. The following procedure was conducted: 100 µL aliquots of calibration standards or serum, 10 µL of internal standard solution (50 µM D6-DMA, 20 µM D7-ADMA, and 100 µM D7-arginine, respectively) and 50 µL of borate buffer (0.025 M Na_2_B_4_O_7_**·**10H_2_O, 1.77 mM NaOH, pH 9.2) were placed into 2.0 mL polypropylene tubes and vortexed (1 min, 25 °C). Derivatization was conducted using 400 µL of acetonitrile (ACN) and 10 µL of 10% BCl in ACN. The solutions were incubated and vortexed (5 min, 25 °C), centrifuged (7 min, 10,000 RPM, 4 °C), and 100 µL of the clear supernatant was transferred into glass vials containing 400 µL of water.

### Conditions of UPLC–ESI–MS Analysis

Analytical chromatography was conducted on Acquity HSS T3 column (50 × 1.0 mm, 1.75 µm) from Waters, using nanoAcquity UPLC system equipped with cooled autosampler (waters). Total run time was 10 min with total flow rate of 250 µL min^−1^ and 0.1% formic acid (FA) in water was used as a mobile phase A and 0.1% FA in methanol as mobile phase B. Sample injection volume was 2 µL. The applied gradient was as follows: 5% B for 0–0.5 min, 5–14% B for 0.5–3 min, 14–60% B for 3–4 min, 60–90% B for 4–4.5 min, 90% B for 4.5–5 min, and 90–5% B for 5–5.10 min.

**Fig. 1:**
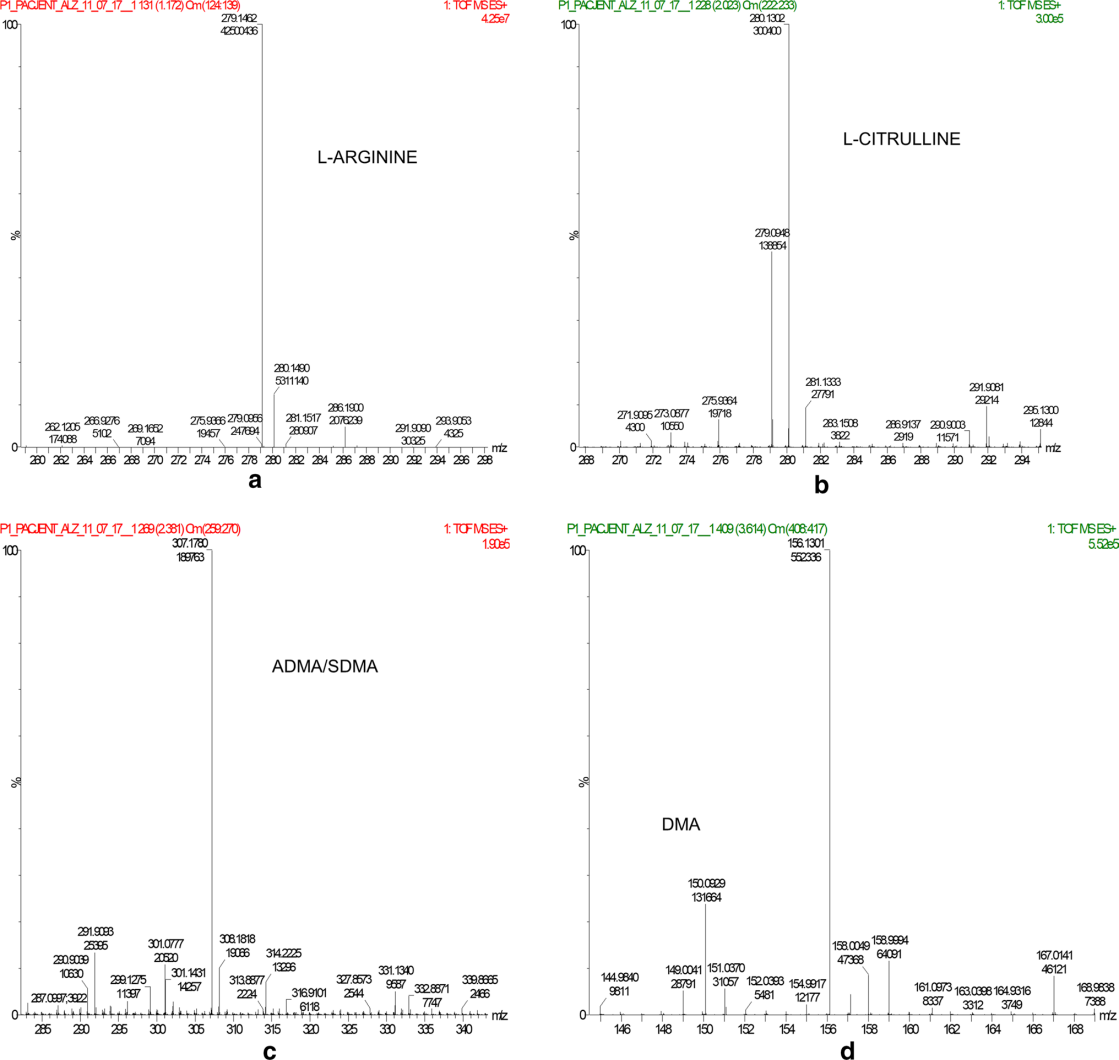
Mass spectra of benzamide derivatives of **a** l-arginine, **b** l-citrulline, **c** ADMA/SDMA, and **d** DMA for serum sample

MS analysis was conducted using Xevo G2 XS Quadrupole TOF MS (waters) with ESI. The spray voltage, source temperature, and the desolvation temperature were set at 0.5 kV, 120 and 450 °C, respectively. Nitrogen was used as the nebulizing and drying gas. Data were acquired by using MassLynx software (waters) for following ions (*m/z*): 279.1457, 286.1749, 307.1717, 314.2076, 280.1297, 150.0919, and 156.1113 for l-arginine, D7-arginine, ADMA, SDMA, D7-ADMA, l-citrulline, DMA, and D6-DMA, respectively (for mass spectrum see Fig. 1).

## Results and Discussion

### Method Development

**Fig. 2:**
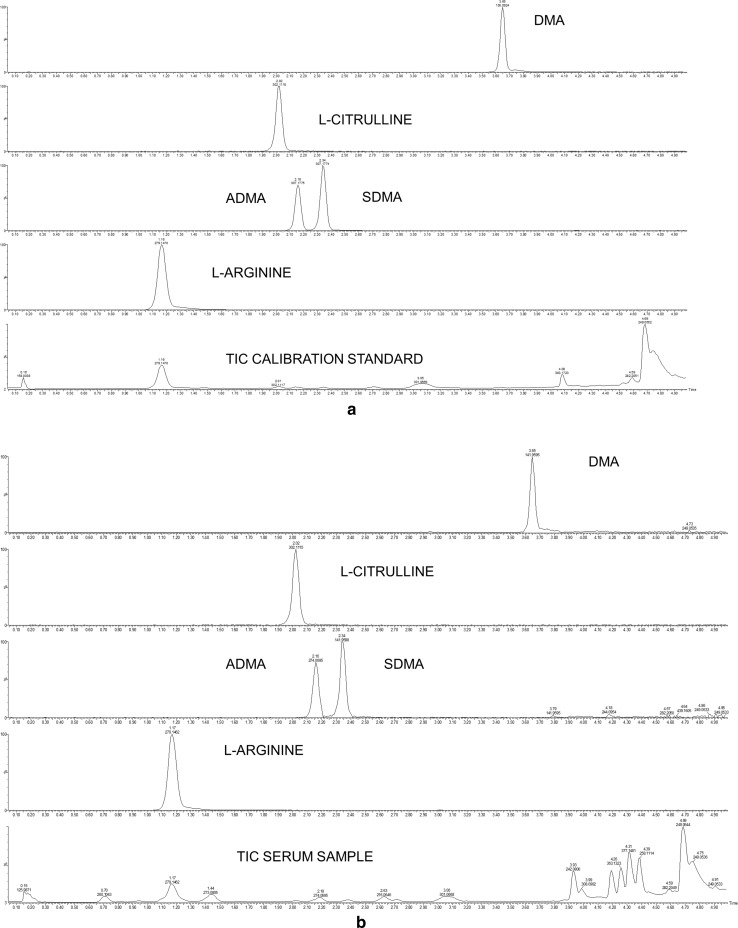
Total ion current (TIC) and extracted ion chromatograms of benzamide derivatives of l-arginine, l-citrulline, ADMA/SDMA, and DMA for **a** calibration standard sample, **b** serum sample

Various analytical methods for the analysis of intermediates and derivatives of NO metabolism have been proposed. However, none of them allows for simultaneous quantitative analysis of l-arginine, l-citrulline, ADMA, SDMA, and dimethylamine. Also chromatographic separation of ADMA and SDMA without derivatization using reversed-phase column was analytically challenging. Previous approaches to chromatographic separation of ADMA and SDMA were conducted via a normal-phase chromatography [20]. Recent paper by Pesek et al. [21], presenting separation of ADMA and SDMA using aqueous normal-phase chromatography, demonstrated the use of in-source fragmentation mass spectrometric technique, in which, however, a complete separation of compounds was not achieved. In order to obtain partial separation, the authors have used an experimental AMPS column; however, the LC method was very long (30 min). Derivatization with benzoyl chloride used in our method, based on reversed-phase chromatography using Acquity UPLC HSS T3 column, promotes retention of analytes on the column. As a result, it enables full chromatographic separation of ADMA and SDMA in a relatively short time (up to 7 min, see Fig. 2).

Butyl derivatives, commonly used in ADMA analysis, do not allow for concomitant determination of DMA concentration [13, 22] and additionally require extended evaporation, which further prolongs sample preparation time. Other commonly used derivatization reagent, *ortho*-phthaldialdehyde (OPA), is mainly used in the analysis with HPLC and fluorescence detection [23, 24]. There have been attempts to use OPA in the analysis on a triple quadrupole mass spectrometer [25]. However, the obtained quantification limits (LOQs) (3.75 µM l-arginine, 0.2 µM ADMA, 0.15 µM SDMA, and 1.25 µM l-citrulline [25]) were worse. In our analytical method, we obtained better LOQs (3.2 µM l-arginine, 0.08 µM ADMA, 0.05 µM SDMA and 1.08 µM l-citrulline; see Table 1). To the best of our knowledge, the very first attempt at simultaneous determination of DMA and ADMA was reported by Tsikas et al. [12]. In their method, none of the procedures led to formation of suitable derivatives of ADMA. Unlike those authors, in our first attempt to quantify DMA together with other NO-associated metabolites, using 2,3,4,5,6-pentafluorobenzoyl chloride as a derivatizing reagent, we failed to obtain suitable DMA derivatives [26]. Change of derivatizing agent to benzoyl chloride as well as replacing sodium carbonate with borate buffer and, consequently, a subtle pH change, improved linearity for DMA derivatives. Of note, we also noticed that a number of available pentafluorobenzoyl chloride reagents were contaminated with DMA what may contribute to the failure of previous method. Compared with the method using pentafluorobenzoyl chloride, the new method additionally allowed for shortening of extraction and derivatization time by half and for reduction in volume of deproteinizing reagent by 20%. Here, we applied ACN as the extraction solvent, which allowed for successful deproteinization and efficient extraction in a single step. The acylation process leading to a benzamide derivative was performed at 25 °C for 5 min. Neither higher temperatures nor longer reaction times visibly affected its performance. However, the efficiency of derivatization was improved by conducting the reaction under alkaline conditions, earlier reported to be optimal [27]. Borate buffer was used to stabilize the pH of the samples and calibration standards. Here, we used a common internal standard, D7-ADMA, for the quantitative determination of ADMA, SDMA, and l-citrulline. It simplified the assay without hampering its performance, as shown by van Dyk et al. [28]. Those authors demonstrated that the addition of standards for l-citrulline and SDMA had no positive effect on assay accuracy or precision. For the separation of samples, we used Acquity HSS T3 column (50 × 1.0 mm, 1.75 µm) with 0.22 µm membrane inline filter (waters). The development of an appropriate mobile phase gradient allowed for complete chromatographic separation of ADMA and SDMA isomers. Exemplary TIC chromatograms of benzamide derivatives of l-arginine, l-citrulline, ADMA/SDMA, and DMA standards and samples obtained using the described method are presented in Fig. 2. Mass spectrometry conditions were optimized to achieve maximum ion generation with abundance.

**Table 1:** Method validation results

Compound	Sensitivity		Linearity		Expected concentration (µM)	Precision and accuracy					
						Within-run (*n* = 5)			Between-run (*n* = 10)		
	LOD (µM)	LOQ (µM)	Regression coefficient (*R*^2^)	Slope CV (%)^a^		Measured concentration (µM)	Precision (CV %)	Accuracy (%)	Measured concentration (µM)	Precision (CV %)	Accuracy (%)
l-Arginine	1.7	3.2	0.998	3.20	5 25 100 200	4.867 ± 0.198 25.49 ± 0.97 99.17 ± 2.68 203.08 ± 4.70	9.14 3.86 1.64 2.32	102.74 98.09 100.83 98.49	5.142 ± 0.035 24.8 ± 01.01 101.85 ± 3.39 199.52 ± 5.44	3.63 4.09 3.33 2.73	97.23 100.87 98.19 100.24
ADMA	0.03	0.08	0.997	3.66	0.05 0.25 1.0 2.5	0.058 ± 0.0001 0.233 ± 0.0003 1.012 ± 0.0004 2.493 ± 0.008	14.88 7.53 2.09 3.52	86.10 107.14 98.80 100.30	0.056 ± 0.0003 0.235 ± 0.022 1.022 ± 0.049 2.477 ± 0.076	10.21 9.42 4.84 3.07	89.29 106.47 97.89 100.93
SDMA	0.02	0.05	0.997	3.33	0.05 0.25 1.0 2.5	0.058 ± 0.00003 0.239 ± 0,0002 0.990 ± 0,002 2.505 ± 0.006	8.99 6.39 3.98 3.20	85.62 104.53 101.03 99.81	0.057 ± 0.00003 0.230 ± 0.016 1.013 ± 0.038 2.506 ± 0.077	9.92 7.11 3.77 3.09	87.72 108.79 98.74 99.75
l-Citrulline	0.36	1.08	0.998	6.12	1 10 20 50	1.036 ± 0.003 10.00 ± 0.07 20.304 ± 0.428 50.019 ± 0.829	5.35 2.58 3.22 1.82	96.53 101.14 98.50 99.96	1.034 ± 0.093 9.88 ± 0.044 20.083 ± 0.617 50.163 ± 1.47	9.01 2.13 3.07 2.93	96.72 101.25 99.58 99.67
DMA	0.06	0.19	0.992	3.89	0.014 0.7 2.8 7.0	0.164 ± 0.00026 0.698 ± 0.001 2.71 ± 0.034 7.19 ± 0.016	9.84 5.49 6.80 1.75	85.32 100.26 103.18 97.30	0.152 ± 0.00004 0.64 ± 0.06 2.61 ± 0.15 7.46 ± 0.19	4.19 9.74 5.56 5.87	92.41 109.69 107.24 93.81

*LOD* limit of detection, *LOQ* limit of quantification, *CV* coefficient of variation

^a^Variability of standard curve slopes is expressed as coefficient of variation

### Method Validation

In practice, it is often difficult or impossible to obtain biological matrices devoid of measured compounds. Therefore, we prepared calibration standards in water. To demonstrate their suitability for analysis, we compared the slopes of calibration curves obtained for standards dissolved in water and serum. The obtained differences in slopes were acceptably low (< 15%), that is, 8.29% for l-arginine, 5.36% for ADMA, 9.72% for SDMA, 6.25% for l-citrulline, and 6.57% for DMA. The established method was validated, and its assay linearity, accuracy and precision, recovery, as well as limits of detection and quantification were determined. If not otherwise stated, all data were averaged from three replicates of five separate surveys. All tests were conducted in water excluding recoveries, which were performed in serum.

#### Calibration Curves and Linearity

Linear regression analysis of relative areas responses vs. concentration was conducted, and regression coefficients (*R*^2^) were calculated. All calibration curves were linear with *R*^2^ > 0.992 and coefficient of variation (CV) of calibration curves slopes at < 6.12%. Respective data are shown in Table 1.

#### Accuracy, Precision, and Stability

Low, medium, and high concentrations of quality control samples (respectively, QC1, QC2, and QC3) were used for determination of accuracy and precision. The results are summarized in Table 1. Stability of the derivatives was tested for QC directly after derivatization procedure (0 h) and after 1 day (24 h). Samples were stored in an autosampler cooled to 6 °C. The results are depicted in Table 2 (presented data are average of five replicates for within-run analysis or of ten replicates for between-run analysis). All calculated CVs were below 15% and as such consistent with FDA guidelines for validation of bioanalytical methods [29].

**Table 2:** Stability of compounds derivatives

Compound	Sample	0 h concentration (µM)	24 h concentration (µM)	Average concentration (µM)	CV (%)
l-Arginine	QC1	4.725	4.333	4.529	8.03
	QC2	105.58	104.92	105.38	1.11
	QC3	196.67	197.19	196.93	3.01
ADMA	QC1	0.0570	0.0527	0.0548	14.65
	QC2	1.088	0.995	1.042	6.99
	QC3	2.512	2.493	2.503	1.83
SDMA	QC1	0.0593	0.0593	0.0593	9.57
	QC2	1.037	1.044	1.041	2.69
	QC3	2.546	2.496	2.521	3.81
l-Citrulline	QC1	1.014	0.982	0.998	4.67
	QC2	19.883	20.249	20.041	2.95
	QC3	50.587	50.308	50.583	3.99
DMA	QC1	0.154	0.150	0.152	3.65
	QC2	0.655	0.604	0.634	9.02
	QC3	7.152	7.513	7.33	5.48

*QC* quality control

#### Limit of Detection and Limit of Quantification

It the current study, the limit of detection (LOD) and the limit of quantification (LOQ) were defined by the standard deviation (SD) obtained for the lowest standard of the calibration curves. Three replicates (*n* = 3) of five surveys (*n* = 5) were used. The following formula was utilized: 3.3 × SD/slope for LOD and 10 × SD/slope for LOQ. The LOD and LOQ values are listed in Table 1.

#### Recovery

Recovery rates were determined using the following formula: (response obtained for spiked serum − response of non-spiked serum)/response of calibration standard diluted in water × 100. Spiked samples were prepared using the following procedure: 25 µL of calibration standard was added to 75 µL of serum, and then derivatization reaction was performed according to the above described method. The mean recovery results are shown in Table 3.

**Table 3:** Mean recovery rates of the method

	Sera measured concentrations mean ± SD (μM)	Added concentration (μM)	Spiked sera measured concentration mean ± SD (μM)	Recovery (%)
l-Arginine	55.337 ± 1.936	61.485 36.741 5.365	116.03 ± 3.51 91.19 ± 2.64 62.42 ± 0.69	98.83 97.54 127.82
ADMA	0.284 ± 0.024	0.593 0.369 0.065	0.88 ± 0.03 0.63 ± 0.01 0.35 ± 0.01	99.41 93.29 96.37
SDMA	0.248 ± 0.016	0.602 0.369 0.067	0.79 ± 0.02 0.56 ± 0.01 0.31 ± 0.01	90.87 86.78 87.00
l-Citrulline	17.617 ± 0.945	12.120 7.152 1.258	31.90 ± 2.82 26.05 ± 3.00 19.98 ± 2.35	118.91 118.26 109.27
DMA	2.128 ± 0.193	7.609 4.517 0.672	10.05 ± 0.35 6.85 ± 0.20 2.98 ± 0.1	103.86 103.29 118.99

*SD* standard deviation

### Method Application

The established method was applied to compare the concentrations of l-arginine, ADMA, SDMA, l-citrulline, and DMA in 73 serum samples of healthy volunteers, who were previously included in studies on patients with schizophrenia [30–32]. The following data were gathered from the above group of participants: age, education, anthropometric parameters (body mass index and waist circumference), and cigarette smoking status assessed using the pack-year index and Fagerström test for nicotine dependence (FTDN) [33]. These subjects had no psychiatric and known somatic health impairments. The results obtained with our method are consistent with other reports of results obtained for l-arginine, ADMA, SDMA, l-citrulline, and DMA, and are 112.84 ± 45.20, 0.38 ± 0.05, 0.33 ± 0.04, 24.54 ± 6.11, and 0.94 ± 0.39 µM, respectively. For intermediates and derivatives of NO metabolism, the reported ranges of concentrations are as follows: for l-arginine 129.5 ± 30 µM [34], for ADMA 0.497 ± 0.063 µM [35], for SDMA 0.526 ± 0.101 µM [35], for l-citrulline 24.3 µM (median 11.6–47.3) [36], and for DMA 3.3 ± 1.5 µM [11]. Detailed characteristics of healthy volunteers are shown in Table 4. Higher individual variability in arginine and DMA, both observed in the current study and reported elsewhere [11], may reflect differences in diet between study participants, as food is an important source of both metabolites. Therefore, the possible effect of diet should be taken into account when interpreting results on arginine and DMA.

**Table 4:** General characteristics and serum levels of metabolites in healthy volunteers

Age	26.11 ± 3.01
Sex, males (%)	44 (60.3)
Education, higher (%)	23 (31.5)
BMI, kg/m^2^	22.95 ± 2.58
Waist circumference (cm)	78.71 ± 10.03
Cigarette smoking, *n* (%)	22 (30.1)
FTND score	1.30 ± 2.29
Pack-year index	1.09 ± 2.38
l-Arginine (μM)	112.84 ± 45.20
Dimethylamine (μM)	0.94 ± 0.39
ADMA (μM)	0.38 ± 0.05
SDMA (μM)	0.33 ± 0.04
l-Citrulline (μM)	24.54 ± 6.11
l-Arginine: ADMA	297.85 ± 126.12

Data expressed as mean ± SD for continuous variables and the number of cases (%) for categorical variables

*FTND* Fagerström test for nicotine dependence

### Statistical Analysis

Correlations between continuous variables were evaluated using the Spearman’s rank correlation test. Effects of sex on metabolic parameters were tested using the Mann–Whitney *U* test. Results were considered as statistically significant if the *p* value was < 0.05. Statistical analysis was performed using the Statistical Package for Social Sciences (SPSS) version 20.

There were no significant correlations between general characteristics of healthy volunteers and metabolic parameters except for a significant negative correlation between serum levels of dimethylamine and age (Table 5).

**Table 5:** Correlations between metabolic parameters and general characteristics of healthy volunteers

	l-Arginine	l-Citrulline	ADMA	SDMA	DMA	l-Arginine: ADMA
Age	*r* = − 0.052	*r* = − 0.188	*r* = − 0.124	*r* = − 0.155	*r* = − 0.274*	*r* = − 0.013
BMI	*r* = − 0.079	*r* = 0.066	*r* = − 0.077	*r* = 0.055	*r* = 0.168	*r* = − 0.067
Waist circumference	*r* = − 0.065	*r* = 0.073	*r* = − 0.172	*r* = 0.298	*r* = 0.208	*r* = 0.014
FTND score	*r* = 0.139	*r* = − 0.044	*r* = 0.051	*r* = − 0.055	*r* = − 0.130	*r* = 0.109
Pack-year index	*r* = 0.156	*r* = − 0.071	*r* = 0.029	*r* = − 0.084	*r* = − 0.098	*r* = 0.144

*p = 0.025; statistical significance for remaining correlation coefficients was not reached

*FTND* Fagerström test for nicotine dependence

Correlations between metabolic parameters are presented in Table 6. There was a significant positive correlation between serum levels of l-arginine, ADMA, and l-arginine:ADMA ratio. Serum l-citrulline levels positively correlated with serum levels of ADMA, SDMA, and dimethylamine. There was a significant positive correlation between serum levels of SDMA and ADMA, as well as between serum levels of dimethylamine and SDMA. Although these analyses were conducted on apparently healthy individuals and therefore the individual variability in measured parameters was relatively low, similar correlation patterns were reported in patients with chronic kidney disease [37]. DMA correlation with SDMA may be result of both metabolites being excreted with urine and reportedly reflect kidney function [11, 38].

**Table 6:** Correlations between metabolic parameters

	l-Arginine	l-Citrulline	ADMA	SDMA	DMA	l-Arginine: ADMA
l-Arginine	–					
l-Citrulline	*r* = 0.221	–				
ADMA	*r* = 0.308^a^	*r* = 0.318^a^	–			
SDMA	*r* = 0.145	*r* = 0.480^b^	*r* = 0.413^b^	–		
DMA	*r* = 0.058	*r* = 0.302^c^	*r* = 0.020	*r *= 0.320^a^	–	
l-Arginine: ADMA	*r* = 0.837^c^	*r* = 0.117	*r* = − 0.199	*r* = − 0.033	*r* = 0.069	–

^a^*p* < 0.01

^b^*p* < 0.001

^c^*p* < 0.05

There was no significant effect of gender on serum levels of metabolic parameters with exception of l-citrulline. Specifically, males had significantly higher levels of l-citrulline (26.03 ± 4.88 vs. 22.34 ± 7.12, *p* = 0.019).

## Conclusion

The liquid chromatography Q-TOF hybrid high-resolution mass spectrometry method applies a process of simultaneous extraction and derivatization of the intermediates and derivatives of NO metabolism using the benzoyl chloride as derivatization reagent. The analyte concentrations obtained with our method were within a range reported by others for serum samples using HPLC analysis or LC–MS/MS technique. To minimize matrix impact on quantitative measurements, a stable internal standard, D7-ADMA, was used for the determination of ADMA, SDMA, and l-citrulline. The use of D7-ADMA allowed us to achieve high accuracy and precision without increasing costs of the analysis. The LC–ESI–QTOF methodology reported here for serum samples is simple, fast, accurate, and precise, and may be useful for the determination of l-arginine, ADMA, SDMA, l-citrulline, and DMA in biological samples. Our approach allows for simultaneous assessment of a panel of intermediates and derivatives of NO metabolism and as such is a time- and cost-saving approach. In addition, simultaneous analysis of several compounds reduces the required amount of biological material compared with the several single-compound analyses, making the method more suitable for application in clinical/diagnostic laboratory. Moreover, our LC–ESI–QTOF method allowed for including DMA in a panel of NO-associated metabolites. Simultaneous determination of DMA and citrulline can be of interest as a better surrogate indicator of DDAH activity than citrulline alone due to citrulline being a coproduct of NOS as well as substrate in arginine synthesis [39, 40]. Owing to its being secreted with urine, DMA accumulation in blood, similar to that of SDMA [41], may reflect impairment of kidney function. Moreover, little is known about pathophysiological role of DMA. Nonetheless, it has been shown that, in the presence of increased NO concentration, an active carcinogen—dimethylnitrosamine—is formed from DMA [42].

## Abbreviations

ACN: Acetonitrile
ADMA: Asymmetric dimethylarginine
BCl: Benzoyl chloride
CV: Coefficient of variation
DDAH: Dimethylarginine dimethylaminohydrolase
DMA: Dimethylamine
ESI: Electrospray ionization source
FTDN: Fagerström test for nicotine dependence
GLC–MS: Gas–liquid chromatography–mass spectrometry
HPLC: High-performance liquid chromatography
LC–MS: Liquid chromatography coupled with mass spectrometry
LOD: Limit of detection
LOQ: Limit of quantification
*m/z*: Mass to charge ratio
MRM: Multiple reaction monitoring
NO: Nitric oxide
NOS: Nitric oxide synthase
OPA: Ortho-phthaldialdehyde
QC: Quality control samples
SDMA: Symmetric dimethylarginine
TFA: Trifluoroacetic acid

## References

[1] Omer N, Rohilla A, Rohilla S, Kushnoor A (2012). Review article nitric oxide: role in human biology. Int J Pharma Sci Drug Res.

[2] Abu-Soud HM, Wang J, Rousseau DL (1995). Neuronal nitric oxide synthase self-inactivates by forming a ferrous–nitrosyl complex during aerobic catalysis. J Biol Chem.

[3] Kopincová J, Púzserová A, Bernátová I (2011). Biochemical aspects of nitric oxide synthase feedback regulation by nitric oxide. Interdiscip Toxicol.

[4] Forstermann U, Sessa WC (2012). Nitric oxide synthases: regulation and function. Eur Heart J.

[5] Ogawa T, Kimoto M, Sasaoka K (1989). Purification and properties of a new enzyme, NG, NG-dimethylarginine dimethylaminohydrolase, from rat kidney. J Biol Chem.

[6] Tsikas D, Böger RH, Sandmann J (2000). Endogenous nitric oxide synthase inhibitors are responsible for the l-arginine paradox. FEBS Lett.

[7] Tsikas D, Sandmann J, Savva A (2000). Assessment of nitric oxide synthase activity in vitro and in vivo by gas chromatography–mass spectrometry. J Chromatogr B Biomed Sci Appl.

[8] Kielstein A, Tsikas D, Galloway GP, Mendelson JE (2007). Asymmetric dimethylarginine (ADMA)—a modulator of nociception in opiate tolerance and addiction?. Nitric Oxide Biol Chem.

[9] Kielstein JT, Fliser D, Veldink H (2009). Asymmetric dimethylarginine and symmetric dimethylarginine: axis of evil or useful alliance?. Semin Dial.

[10] Lamglait B, Vandenbunder-Beltrame M (2017). Evaluation of symmetric dimethylarginine as an early biomarker of chronic kidney disease in captive cheetahs (*Acinonyx jubatus*). J Zoo Wildl Med.

[11] Teerlink T, Hennekes MW, Mulder C, Brulez HF (1997). Determination of dimethylamine in biological samples by high-performance liquid chromatography. J Chromatogr B Biomed Sci Appl.

[12] Tsikas D, Thum T, Becker T (2007). Accurate quantification of dimethylamine (DMA) in human urine by gas chromatography–mass spectrometry as pentafluorobenzamide derivative: evaluation of the relationship between DMA and its precursor asymmetric dimethylarginine (ADMA) in health and disease. J Chromatogr B Anal Technol Biomed Life Sci.

[13] Schwedhelm E, Tan-Andresen J, Maas R (2005). Liquid chromatography–tandem mass spectrometry method for the analysis of asymmetric dimethylarginine in human plasma. Clin Chem.

[14] D’Apolito O, Paglia G, Tricarico F (2008). Development and validation of a fast quantitative method for plasma dimethylarginines analysis using liquid chromatography–tandem mass spectrometry. Clin Biochem.

[15] Martens-Lobenhoffer J, Bode-Böger SM, Clement B (2016). First detection and quantification of Nd-monomethylarginine, a structural isomer of NG-monomethylarginine, in humans using MS3. Anal Biochem.

[16] Gopu CL, Hari PR, George R (2013). Simultaneous determination of homocysteine and asymmetric dimethylarginine in human urine by liquid chromatography-tandem mass spectrometry. J Chromatogr B Anal Technol Biomed Life Sci.

[17] Martens-Lobenhoffer J, Bode-Böger SM (2012). Quantification of l-arginine, asymmetric dimethylarginine and symmetric dimethylarginine in human plasma: a step improvement in precision by stable isotope dilution mass spectrometry. J Chromatogr B Anal Technol Biomed Life Sci.

[18] Zotti M, Schiavone S, Tricarico F (2008). Determination of dimethylarginine levels in rats using HILIC–MS/MS: an in vivo microdialysis study. J Sep Sci.

[19] Paglia G, D’Apolito O, Tricarico F (2008). Evaluation of mobile phase, ion pairing, and temperature influence on an HILIC–MS/MS method for l-arginine and its dimethylated derivatives detection. J Sep Sci.

[20] Vicente FB, Vespa G, Miller A, Haymond S (2016). Quantification of arginine and its methylated derivatives in plasma by high-performance liquid chromatography tandem mass spectrometry (LC–MS/MS). Methods Mol Biol.

[21] Pesek JJ, Matyksa MT, Modereger B (2016). The separation and analysis of symmetric and asymmetric dimethylarginine and other hydrophilic isobaric compounds using aqueous normal phase chromatography. J Chromatogr A.

[22] Boelaert J, Schepers E, Glorieux G (2016). Determination of asymmetric and symmetric dimethylarginine in serum from patients with chronic kidney disease: UPLC–MS/MS versus ELISA. Toxins (Basel).

[23] Dobashi Y, Santa T, Nakagomi K, Imai K (2002). An automated analyzer for methylated arginines in rat plasma by high-performance liquid chromatography with post-column fluorescence reaction. Analyst.

[24] Markowski P, Baranowska I, Baranowski J (2007). Simultaneous determination of l-arginine and 12 molecules participating in its metabolic cycle by gradient RP-HPLC method. Application to human urine samples. Anal Chim Acta.

[25] Martens-Lobenhoffer J, Bode-Böger SM (2003). Simultaneous detection of arginine, asymmetric dimethylarginine, symmetric dimethylarginine and citrulline in human plasma and urine applying liquid chromatography–mass spectrometry with very straightforward sample preparation. J Chromatogr B Anal Technol Biomed Life Sci.

[26] Wisniewski J, Fleszar MG, Piechowicz J (2017). A novel mass spectrometry-based method for simultaneous determination of asymmetric and symmetric dimethylarginine, l-arginine, and l-citrulline optimized for LC–MS–TOF and LC–MS/MS. Biomed Chromatogr.

[27] Lundblad RL (1994). Techniques in protein modification.

[28] van Dyk M, Mangoni AA, McEvoy M (2015). Targeted arginine metabolomics: a rapid, simple UPLC–QToF–MSE based approach for assessing the involvement of arginine metabolism in human disease. Clin Chim Acta.

[29] FDA F and DA, Food and Drug Administration (2001) Guidance for industry: bioanalytical method validation.Freedom of Information Staff, (HFI 35). Food and Drug Administration, Rm. 12 A - 30, 5600 Fishers Lane, Rockville, MD 20857, USA, http://www.fda.gov/

[30] Misiak B, Frydecka D, Slezak R (2014). Elevated homocysteine level in first-episode schizophrenia patients—the relevance of family history of schizophrenia and lifetime diagnosis of cannabis abuse. Metab Brain Dis.

[31] Misiak B, Łaczmański Ł, Słoka NK (2016). Metabolic dysregulation in first-episode schizophrenia patients with respect to genetic variation in one-carbon metabolism. Psychiatry Res.

[32] Misiak B, Szmida E, Karpiński P (2015). Lower LINE-1 methylation in first-episode schizophrenia patients with the history of childhood trauma. Epigenomics.

[33] Pomerleau CS, Majchrzak MJ, Pomerleau OF (1989). Nicotine dependence and the Fagerstrom Tolerance Questionnaire: a brief review. J Subst Abuse.

[34] Psychogios N, Hau DD, Peng J (2011). The human serum metabolome. PLoS ONE.

[35] Teerlink T (2007). HPLC analysis of ADMA and other methylated l-arginine analogs in biological fluids. J Chromatogr B Anal Technol Biomed Life Sci.

[36] Ruoppolo M, Campesi I, Scolamiero E (2014). Serum metabolomic profiles suggest influence of sex and oral contraceptive use. Am J Transl Res.

[37] El-Sadek AE, Behery EG, Azab AA (2016). Arginine dimethylation products in pediatric patients with chronic kidney disease. Ann Med Surg.

[38] Kielstein JT, Salpeter SR, Bode-Boeger SM (2006). Symmetric dimethylarginine (SDMA) as endogenous marker of renal function—a meta-analysis. Nephrol Dial Transplant.

[39] Tain YL, Hsieh CS, Lin IC (2010). Effects of maternal l-citrulline supplementation on renal function and blood pressure in offspring exposed to maternal caloric restriction: the impact of nitric oxide pathway. Nitric Oxide Biol Chem.

[40] Shin S, Fung SM, Mohan S, Fung HL (2011). Simultaneous bioanalysis of l-arginine, l-citrulline, and dimethylarginines by LC–MS/MS. J Chromatogr B Anal Technol Biomed Life Sci.

[41] Fleck C, Schweitzer F, Karge E (2003). Serum concentrations of asymmetric (ADMA) and symmetric (SDMA) dimethylarginine in patients with chronic kidney diseases. Clin Chim Acta.

[42] Zhang AQ, Mitchell SC, Smith RL (1995). Dimethylamine in human urine. Clin Chim Acta.

